# Extracellular Vesicle-Mediated Communication Within Host-Parasite Interactions

**DOI:** 10.3389/fimmu.2018.03066

**Published:** 2019-01-15

**Authors:** Zhenyu Wu, Lingling Wang, Jiaying Li, Lifu Wang, Zhongdao Wu, Xi Sun

**Affiliations:** ^1^Department of Parasitology of Zhongshan School of Medicine, Sun Yat-sen University, Guangzhou, China; ^2^Key Laboratory of Tropical Disease Control (SYSU), Ministry of Education, Guangzhou, China; ^3^Provincial Engineering Technology Research Center for Biological Vector Control, Guangzhou, China

**Keywords:** parasite, extracellular vesicle, intercellular communication, parasite-host interaction, exosome

## Abstract

Extracellular vesicles (EVs) are small membrane-surrounded structures released by different kinds of cells (normal, diseased, and transformed cells) *in vivo* and *in vitro* that contain large amounts of important substances (such as lipids, proteins, metabolites, DNA, RNA, and non-coding RNA (ncRNA), including miRNA, lncRNA, tRNA, rRNA, snoRNA, and scaRNA) in an evolutionarily conserved manner. EVs, including exosomes, play a role in the transmission of information, and substances between cells that is increasingly being recognized as important. In some infectious diseases such as parasitic diseases, EVs have emerged as a ubiquitous mechanism for mediating communication during host-parasite interactions. EVs can enable multiple modes to transfer virulence factors and effector molecules from parasites to hosts, thereby regulating host gene expression, and immune responses and, consequently, mediating the pathogenic process, which has made us rethink our understanding of the host-parasite interface. Thus, here, we review the present findings regarding EVs (especially exosomes) and recognize the role of EVs in host-parasite interactions. We hope that a better understanding of the mechanisms of parasite-derived EVs may provide new insights for further diagnostic biomarker, vaccine, and therapeutic development.

## Introduction

To date, parasitic diseases remain a notable worldwide problem threatening human health; such diseases include schistosomiasis, malaria, toxoplasmosis, leishmaniasis, and trichomoniasis. Since these diseases mainly occur in developing countries and poor regions, they are somewhat neglected, although they burden many people, and cause multiple deaths every year. In addition, the problem of drug resistance has led to growing concerns due to the overuse of antiparasitic medicines (1). As a result, increasing awareness of parasites and finding better ways to cope with parasitic infections remain imperative. During long-term coevolution with different host species, parasites can adopt complex strategies of communication, manipulate, and even hijack the host immune system, and generally exhibit an ability to drive a physiological and immunological homeostasis that benefits their survival in the host (2). Thus, it is particularly important to recognize how parasites influence immune status through cell-cell communication and promote their survival in hosts to achieve long-term parasitism. Previous studies have mainly focused on the secretion of parasitic signaling molecules, such as proteins, glycans, lipids, and nucleic acids, involved in intercellular communication and promoting the host Th2 immune response to modulate the body's immune functions (3). However, long-term studies have not found a single parasite-derived substance that plays a decisive role in parasite-host interactions. A parasite is more likely to release these modulatory molecules in a variety of “packages” and express a dominant function (4). In recent years, with the discovery of extracellular vesicles (EVs) and their importance in cellular crosstalk, researchers have focused their attention on EVs in parasitic diseases.

EVs are small membrane vesicles derived from the endocytic compartment of different kinds of cells such as reticulocytes, karyocytes, and platelets, which are present in most human bodily fluids, including blood, cerebrospinal fluid (CSF), sputum, saliva, ascites, amniotic fluid, bile, semen, breast milk, and urine (5). As pockets, EVs carry a series of bioactive cargos consisting of lipids, proteins, metabolites, DNA, and RNA (mRNA, miRNA, and ncRNA) and can be divided into four important types according to the mechanism of their generation and their size: (1) exosomes, (2) microvesicles, (3) apoptotic bodies, and (4) oncosomes (Table 1) (9).

**Table 1:** Comparison and classification of EVs according to their mechanism of generation.

**Classification of EVs**	**Exosomes**	**Microvesicles**	**Apoptotic bodies**	**Oncosomes**
Origin	Interior budding of the endosomal membrane to form large MVBs	Extracellular membrane yielding particles	Large-scale plasma membrane blebbing, released during apoptotic cell death (6)	Larger size EV population from highly migratory cancer cells (7, 8)
Size	30–150 nm	100–1,000 nm	1,000–5,000 nm	1–10 μm
Specific markers	CD63, CD9, Alix HSP70, TSG101,	Annexin V, flotillin-2, CD40, integrin, metalloproteinase	Annexin V, DNA histone	Cav-1
Separation method	Ultracentrifugation, gradient ultracentrifugation, precipitation solution, size exclusion chromatography (SEC) column purification, and microfluidic chip	Ultracentrifugation	No standardized approach	No standardized approach

Exosomes are the smallest EVs but have attracted attention from scientists (10). Exosomes measure between 40 and 100 nm, have a density between 1.13 and 1.19 g/mL, and consist of cytoplasm enclosed in a lipid bilayer with transmembrane proteins exposed to the extracellular environment (11). Exosomes are made up through the fusion of multivesicular bodies (MVBs) and intraluminal vesicles (ILVs) (12). Exosome release provides a mechanism of intercellular communication, and specific exosome functions depend on the derived-cell type and composition. More specifically, exosomes play roles in protein secretion, antigen presentation, shuttling of RNAs and infectious agents, and pathogen immune surveillance; furthermore, exosomes can even be used as vaccine candidates and biomarkers for the diagnosis and treatment of diseases. It is worth noting that not only parasite-derived exosomes but also exosomes released from host cells after parasite invasion are involved in the pathogenic mechanisms of parasitic diseases (13). On one hand, exosomes released from a parasite contain conserved parasite-specific information, including proteins, RNA, ncRNA, and nucleic acids, which are transferred to the host cells and can then modulate the host immune system, participate in immune escape by the parasite, and ultimately promote infection. On the other hand, when suffering from the stress of a parasitic infection, host cells also release exosomes that activate immune cells such as NK cells, macrophages, monocytes, T cells and B cells and play anti-infection roles. Thus, in this review, we give a comprehensive summary of EVs, especially exosomes, and focus mainly on their bidirectional regulatory effects on parasitic infections. Essential information about parasitic exosomes and exosomal molecules in this article is summarized in Table 2.

**Table 2:** Essential information on various exosomes and exosomal molecules in different parasitic diseases.

**Disease**	**Pathogen**	**Exosome type/molecule**	**Cell origin**	**Functions**	**Mechanism**	**Potential applications**	**References**
Malaria	*P. falciparum*	Exosome-like vesicle	iRBC	Promote malaria transmission and parasite survival	Intercellular communication via gene delivery	Target of malaria therapeutics	(14)
	*P. yoelii*	Exosome	Infected reticulocyte	Induce reticulocytosis and a protective immune response	Antigen presentation by exosomes with parasite proteins	Malaria vaccine	(15)
		Exosome	iRBC	Inhibit angiogenesis and tumor growth	miRNAs in exosomes inhibit VEGFR2 expression in endothelial cells	Anticancer drug	(16)
Leishmaniasis	*L. donovani*	Exosome	Parasite	Inhibit the macrophage immune response	Induce macrophages to secrete IL-8 rather than TNF-α	Unknown	(17)
		Exosome	Parasite	Inhibit the immune responses of monocytes and monocyte-derived DCs	Facilitate IL-10 production and dampen TNF-α activation to inhibit the monocyte immune response to IFN-γ; reduce monocyte-derived DC production of inflammatory cytokines such as TNF-α, IL-10 and IL-12p70	Vaccine adjuvant for leishmaniasis	(18)
		CPN10	Parasite	Inhibit parasite survival but simultaneously dampen the macrophage immune response	Inhibit *Leishmania* uptake by macrophages but negatively regulate macrophage immunity-related proteins	Unknown	(19)
	*L. mexicana*	Exosome	Infected macrophages	Modulate the host immune response and promote *Leishmania* survival	Activate signal molecules and immune-related DNA	Unknown	(20)
		EF-1α	Parasite	Modulate the host immune response and promote *Leishmania* survival	Activate PTPs, modulate IFN-γ signaling and inhibit macrophage reactions, including the production of TNF-α and NO	Unknown	(21)
	*L. major*	GP63^*^	Parasite	Immune modulation, exacerbation of the *Leishmania* infection and exosome formation	Regulate PTPs and TFs in macrophages; inhibit IL-1β production via inhibition of the NLRP3 inflammasome; cleave Dicer1 and reduce miRNA-122 production and serum cholesterol; modulate *Leishmania* exosome cargo sorting	Potential therapeutic target	(22)
		LmPRL-1	Parasite	Promote parasite survival in the host and induce protective immunity	Unknown	Unknown	(23)
		LieIF	Parasite	Inhibit *Leishmania* growth	Promote NO and ROS production with or without activation of MIP-1a and TNF-α	Potential therapeutic molecule	(24)
Toxoplasmosis	*T. gondii*	Exosome	Infected DCs	Induce protective immunity against *T. gondii* infection	Prompt the proliferation of splenocytes with the enrichment of Th1 cytokines such as IL-2 and IFN-γ and reduced expression of Th2 cytokines such as IL-5 and IL-10	Vaccine preparation	(25)
		Exosome	Infected SRDCs	Induce protective immunity against *T. gondii* infection	Induce a strong cellular response and humoral response, including the increased production of serum antibodies and IgA antibodies in the intestinal tract	Vaccine preparation	(26)
		Exosome	Infected macrophages	Induce protective immunity against *T. gondii* infection	Exosomes contain PAMPs as a crucial mechanism for immune surveillance in a TLR- and MyD88-dependent manner	Vaccine preparation	(26)
		Exosome-like vesicle	Infected human foreskin fibroblasts	Most likely mediating neurologic effects in the *T. gondii* infection	The exosome is full of mRNA, including thymosin beta 4, eukaryotic translation EF-1α, Rab-13 and LLP homolog, which were previously described to mediate neurologic activity	Unknown	(27)
		Exosome	Infected cells	L6 cells present alterations in the cell cycle and cell proliferation and retention at the S or G2/M cell phase	11 miRNAs in exosomes were supposed to regulate the expression of host cell genes	Unknown	(28)
		Exosome	Parasite	Induce protective immunity against *T. gondii* infection	Promote macrophage activation with increased production of IFN-γ, IL-12 and TNF-α; moreover, both humoral and cellular immune responses are stimulated in this process	Vaccine preparation	(29)
Trichomoniasis	*T. vaginalis*	Exosome	Parasite	Facilitate *T. vaginalis* invasion and modulate host inflammatory activation	Promote pathogen adherence to epithelial cells; inhibit IL-8 secretion by ectocervical cells and neutrophil migration to the infection site	Potential use in trichomoniasis diagnosis and treatment	(30)
		Exosome-like vesicle	Parasite	Modulate the host immune response and dampen the inflammatory reaction	Promote IL-10 production and inhibit the expression of immune cytokines such as IL-6, IL-13 and IL-17	Unknown	(31)
	*T. brucei*	Exosome	Parasite	Play an important role in pathogenic processes and parasite nutrient supply	Mediate the release of most excreted/secreted proteins	Unknown	(32, 33)
		Exosome	Parasite	Modulate *Trypanosome* social motility	Keep pathogens away from injured cells and inappropriate environments	Unknown	(34)
		Exosome	Parasite	Modulate rRNA, snoRNA and mRNA processing and quality control	Unknown	Unknown	(35–37)
	*T. cruzi*	Exosome	Parasite	Promote the transformation of *T. cruzi* from the epimastigote to the trypomastigote form and increase host cell susceptibility by inducing changes in the expression profiles of some genes	Exosome-contained tsRNAs can be passed on to other pathogen and host cells	Unknown	(38)
Schistosomiasis	*S. japonicum*	Exosome-like vesicle	Parasite	Mediate parasite-host communications and activate the host immune response	Promote M1 macrophage polarization with increased production of pro-inflammatory factors	Potential use of diagnostic markers, new vaccines, and therapies for schistosomiasis	(39)
		Exosome-like vesicle	Parasite	Deliver miRNA to mammalian cells	Mediate parasite-host interactions	Unknown	(40)
		Exosome	*Schistosoma japonicum* SEA-pulsed DCs	Attenuate the severity and repress the progression of IBD	Reduce the production of pro-inflammatory cytokines and promote the production of anti-inflammatory cytokines via an unknown mechanism	Immunosuppressive drug	(41)
	*S. mansoni*	Exosome	Parasite	Play important roles in host-parasite interactions	Not clear	Vaccines and therapeutics	(42)
		EVs	Sera of infected patients	New tool for diagnosing schistosomiasis	The 2 highest expressed miRNAs in the EVs has the highest sensitivity and specificity.	Diagnosis method	(43)
Lymphatic filariasis	*B. malayi*	Exosome-like vesicle	Parasite	Plays an important role in early infection; regulate the host immune response	Deliver proteins and small RNA species; M1 macrophage polarization	New targets for disease intervention and diagnosis	(44)
Fascioliasis	*F. hepatica*	Exosome-like vesicle	Parasite	Parasite-host communications	Exosome-like vesicles are internalized by intestinal cells	Unknown	(45, 46)
Rodent intestinal infection	*H. polygyrus*	Exosome	Parasite	Inhibit a type 2 innate response and eosinophilia activation, subsequently repressing the host immune response; 9- to 62-fold increase in plasmalogen content and a relative lack of sphingomyelin and cholesterol in *H. polygyrus* exosomes	miRNA and Y RNA in exosomes suppress the expression of genes related to inflammatory reactions such as dampening the activation of IL-33 and DUSP1; increase membrane rigidity and stability	Potential use of immunomodulatory treatment	(47)
Cystic echinococcosis	*Echinococcus granulosus*	Exosome	Parasite	Have an important effect on macrophages and the interplay between parasites and hosts	Exosomes have highly immunogenic and tolerogenic antigens and peptidases.	Unknown	(48)
					Exosomes can reduce the production of NO but do not affect IL-10		
Parasitic gastroenteritis	*Teladorsagia circumcincta*	Exosome-like vesicle	Parasite	Affect host immunity	Proteins in exosome-like vesicles can be bound by IgA and IgG	Potentially key for vaccine development and production	(49)

## Background on Exosomes

### Exosome Composition

The composition of exosomes varies depending on the originating cell type. However, all exosomes share many common protein components (50). Exosomes contain different proteins including Rabs, which cause exosome docking and fusion of the exosome membrane (51). Annexins and the lysobisphosphatidic acid (LBPA)-binding protein Alix may regulate membrane cytoskeleton dynamics and participate in vesicle formation, respectively (52). Heat shock proteins (HSPs) 70 and 90 can promote major histocompatibility complex (MHC)-I and MHC-II to cargo some peptides (53). Several adhesion molecules, such as CD146, CD9, CD18, and CD11, are also carried by exosomes (54, 55). In addition, the thioredoxin peroxidase II and galectin 3 proteins are involved in apoptosis. Tetraspanin markers, including CD9, CD63, CD81, and CD82, are important characteristic features of exosomes.

Lipids are essential components of exosomal membranes. Several studies have shown that exosomes contain 2–3 times the enrichment of cholesterol, SM, and glycosphingolipids relative to that in their parent cells (56). Moreover, lipid content also varies among different cell origins. For example, in mast cell-derived exosomes, the typical lipid composition includes lysophosphatidylcholine, sphingomyelin, phosphatidylcholine, phosphatidylethanolamine, phosphatidylserine, cholesterol, and diglyceride (56, 57). However, the ratios of these lipids in different cell types are different. Additionally, researchers have reported the presence of lipid rafts containing different acylated proteins, such as glycosyl-phosphatidylinositol-anchored proteins and tyrosine kinases of the Src family, on exosomes (58).

Furthermore, mRNAs, tRNAs, rRNAs, rRNA fragments such as natural antisense RNAs; microRNAs (miRNAs); small noncoding RNAs including piRNAs, snRNAs, snoRNAs, scaRNAs, and Y RNAs; and long noncoding RNAs have also been found in exosomes (59, 60). Single-stranded DNA, retrotransposon RNA transcripts, mitochondrial DNA, and oncogene amplifications have been detected in microvesicles, and previous studies have provided evidence that tumor-derived exosomes carry double-stranded DNA (61–63).

### Exosome Biogenesis

Exosomes are membranous compartments that include various ILVs in MVBs for cargo degradation into lysosomes or for secretion as exosomes into the extracellular environment (64). The generation of exosomes is initiated in early endosomes upon endocytosis of extracellular material at the plasma membrane by intraluminal vesicle (ILV) formation, resulting in MVB formation (65). MVBs are intermediate but well-defined compartments that are formed from endosomes via invagination of the limiting endosomal membrane. The mechanisms for protein sorting to MVBs consist of target ubiquitination and preferential aggregation (12, 66). In addition, a key player in MVB biogenesis is the hetero-oligomeric protein complex, which is an endosomal-sorting complex required for transport (ESCRT). ESCRT-I, ESCRT-II, and ESCRT-III recognize monoubiquitinated cargos and promote their inclusion in MVBs (12, 56, 66, 67). Once MVBs are formed, their fusion with the plasma membrane is mediated by the cytoskeleton, fusion machinery such as SNARE proteins, and molecular switches such as low-molecular-weight GTPases. Upon fusion with the plasma membrane, the MVBs are released as exosomes (68).

### Exosome Functions

Distinct functions of exosomes depend on their cellular origin. The roles of exosomes in immunoregulation can be summarized as antigen presentation, modulation of the host immune response, expression of some activated molecules, or complement factors causing immune surveillance and enhancing tumor cell invasion, and mediation of intercellular communication (69, 70).

In infectious diseases, several studies have demonstrated that pathogens such as viruses and prions can exploit exosomes to transfer some pathogen-derived molecules to host cells and cause immune evasion and virus spread (70, 71). In addition, pathogen-infected cells, or the pathogens themselves, release microvesicles or exosomes that can provide antigens to APCs and modulate both the innate and adaptive immune responses. For example, exosomes containing HIV Nef or *Leishmania* GP63 can block T cell activation and induce the apoptosis of certain immune effector cells. In some cases, this process benefits the pathogen, whereas in other cases, the host is benefited (15, 72).

## Function of EVs in Parasite Diseases

### EVs in Malaria

Malaria is a common parasitic disease caused by *Plasmodium* strains, whose clinical features include intermittent fever, vomiting, fatigue, and headache due to damage to erythrocytes. The extensive use of precautionary measures and various antimalarial drugs has decreased the morbidity and mortality of malaria dramatically, but to date, the disease still causes 2,000 human deaths every day (73). Previous research has shown that an increased level of circulating EVs is associated with malaria severity (74). Combes et al. found that knocking out the ABCA1 gene in a mouse model resulted in a decrease in EV production, protecting infected mice from cerebral malaria (75). These studies first demonstrated the importance of EVs in malaria. A pioneering study confirmed that microparticles derived from *Plasmodium berghei*-infected serum contained parasite antigens, which stimulated macrophage CD40 surface expression and TNF release in a dose-specific manner and induced remarkable immune responses via macrophage activation (76). Researchers isolated exosomes from the peripheral blood of BALB/c mice infected by reticulocyte-prone non-lethal *Plasmodium yoelii*, and proteomics revealed that these exosomes contained parasite proteins. Furthermore, immunization of mice with the abovementioned exosomes elicited IgG antibody production, inducing reticulocytosis, and a protective immune response (15). Due to their immunoregulatory functions, exosomes from *Plasmodium* types may be used as components in a malaria vaccine. In addition to regulating immune reactions, exosomes can mediate intercellular communication via nucleic acid delivery. It was reported that the exosome-like vesicles secreted by *Plasmodium falciparum*-infected red blood cells (iRBCs) can deliver DNA among themselves, resulting in an increased number of *P. falciparum* gametocytes and easier transmission to mosquito vectors (77, 78). Moreover, communication via exosome-like vesicles is beneficial to parasite survival in various situations such as stress or drug pressure. The *P. falciparum*-derived protein PfPTP2 is important in this process, and the disruption of PfPTP2 function reduces exosome-like vesicle production (78). Moreover, the exosome-like vesicles may be a potential therapeutic target to block parasite communication and reduce *Plasmodium* transmission. Immunization with *P. yoelii-*infected iRBC-derived EVs can be successfully used against lethal infection (15). Moreover, compared to stimulation by standard adjuvant vaccination, the administration of synthetic microparticles/microspheres loaded with antigens (e.g., merozoite surface protein-1, apical membrane antigen-1, or circumsporozoite protein) of *Plasmodium vivax* through the intranasal mucosal route can improve humoral and cell-mediated immune responses (79, 80). Furthermore, it was reported that exosomes from *P. yoelii-*infected plasma were capable of inhibiting the growth of Lewis lung cancer in a murine model. The researchers detected that a high level of miRNA in exosomes correlated with decreased VEGFR2 expression, thus restraining vessel formation in endothelial cells and ultimately leading to suppressed angiogenesis and tumor growth (16).

On the other hand, accumulating evidence suggests that host-origin EVs are related to the clinical symptoms and severity of malaria. The trans-bilayer distribution of phosphatidylserine at the outer leaflet of the plasma membrane can be modulated by the ATP-binding cassette transporter A1 (ABCA1). ABCA1 knockout mice, which have a lower ability to produce EVs, can significantly reduce the inflammatory response and relieve the severity of cerebral malaria (75). In human patients infected with *P. falciparum*, ABCA1 promoter haplotypes also influence the severity and complications of malaria (81). Moreover, many studies have reported that EV levels will increase during active *Plasmodium* infection. A significant increase in circulating EVs, including EVs originating from platelets, erythrocytes, and endothelial cells, has been shown in *P. falciparum-*infected patients with cerebral malaria in Cameroon and in India (82, 83). This relationship between active infection and increased EV release was also found in *P. vivax-*infected individuals in Brazil. A higher level of platelet-derived EVs correlated with high fever, suggesting that EV levels may play an important role in the inflammatory symptoms of *P. vivax* (84). These reports indicate that EVs may have applications as biomarkers of malaria severity.

### EVs in Leishmaniasis

Leishmaniasis, a parasitic disease caused by the *Leishmania* genus (usually by *Leishmania donovani, Leishmania infantum*, or *Leishmania chagasi*) and spread through sandflies, can be divided into three types according to clinical features: cutaneous, mucocutaneous, and visceral leishmaniasis. Although it sickens millions of people in ~98 countries and causes 2 million new cases as well as 20–50 thousand deaths every year, leishmaniasis remains neglected because it mainly occurs in developing countries and poor regions (85, 86). Silverman et al. first identified 151 proteins that were apparently secreted by *L. donovani* through secretome analysis; interestingly, only 14% of these proteins were targeted for export and consisted of a classic eukaryotic amino-terminal secretion signal peptide. In contrast, a large number of eukaryotic exosome proteins were found, which suggested a vesicle-based pathway in *Leishmania-*derived intercellular communication (86). Sequentially, 329 *Leishmania* proteins have been detected and account for more than 52% of the general proteins secreted by *Leishmania*, which confirms vesicle-based secretion as the major protein secretion mechanism of *Leishmania*. Interestingly, changes in temperature and pH modulate the abundance and composition of *Leishmania* exosomes, and this phenomenon probably reflects different packages of virulence factors in different environments. In addition, the uptake of exosomes induces macrophages to secrete IL-8 rather than TNF-α through an unknown mechanism. This finding suggests that *Leishmania* exosomes function similarly to mammalian exosomes in long-range communication and immune modulation (17). Studies by Silverman et al. were the first to affirm exosome-based secretion in protozoans. *Leishmania* releases exosomes to modulate immune reactions, and macrophages also secrete exosomes to affect *Leishmania* organisms. Through a comparative proteomic and functional analysis, *Leishmania mexicana* was discovered to change the functions and targets of exosomes secreted by macrophages, resulting in the activation of signal molecules and immune-related DNA in a murine model (20). Recently, the role of exosomes in *Leishmania*-host interactions has been investigated in more detail. Similar to whole parasites, *Leishmania* exosomes can strongly influence macrophage cell signaling and function and were shown to be pro-inflammatory and to have the ability to recruit neutrophils at the inoculation site, exacerbating the resulting pathology (87). *Leishmania* exosomes may also impact the immune reactions of monocytes and dendritic cells (DCs) in addition to those of macrophages and neutrophils (88). Exosomes from *Leishmania donovani* facilitate IL-10 production and dampen TNF-α activation to inhibit the monocyte immune response to IFN-γ. In addition, monocyte-derived DCs are negatively regulated by exosomes, presenting decreased production of some inflammatory cytokines such as TNF-α and IL-12p70. Intriguingly, exosomes from *L. donovani* lacking HSP100 promote pro-inflammatory activation and protective immunity rather than suppressing the immune system, suggesting a significant role for HSP100 in exosomal protein sorting. Currently, one reason why vaccine preparations for *Leishmania* are problematic may be the deficiency of applicable adjuvants (18). Furthermore, *Leishmania* exosomes, as a kind of lipid adjuvant, may potentially be useful for vaccine preparations. It is worth mentioning that *Leishmania* exosomes are also related to antimony resistance (89).

Since many studies of *Leishmania* exosomes concern exosomal molecules, here, we focus on the descriptions of these molecules. *Leishmania* GP63, which is found on exosomes, is a zinc metalloprotease fixed on the surface of promastigotes by a GPI anchor. GP63 has been shown to play an important role in modulating the immune response of macrophages through the regulation of protein tyrosine phosphatases (PTPs) and transcription factors (TFs) (90). Studies have investigated differences in the macrophage immune reaction and exosome constitution between *Leishmania major* (WT) and *L. major* gp63-/- (KO). KO *Leishmania* presented a prominently decreased modulatory capacity and a large change in exosome protein sorting relative to WT *Leishmania*, which implied the critical role of GP63 in immune modulation and exosome formation (22). GP63 from *L. donovani* exosomes also cleaved the nuclease Dicer1 and consequently reduced miRNA-122 production, leading to a decreased level of serum cholesterol, and exacerbation of *Leishmania* infection (91). A recent study demonstrated that GP63 from *L. mexicana* exosomes decreases IL-1β production via inhibition of NLRP3 inflammasomes to suppress the host immune response (89). Another protein, elongation factor-1α (EF-1α), was also detected in *Leishmania* exosomes. EF-1α induces the activation of PTPs, leading to the negative modulation of IFN-γ signaling, and the inhibition of macrophage reactions, including the production of TNF-α and NO (21). A novel PRL-like phosphatase, LmPRL-1, was detected in *L. major* exosomes and was found to contribute to parasite survival in macrophages via an unknown mechanism (23). Another novel exosomal protein, chaperonin 10 (CPN10), which is released by *L. donovani*, was recently discovered to inhibit *Leishmania* uptake by macrophages and negatively regulate macrophage immunity-related proteins. Interestingly, it seems paradoxical that CPN10 inhibits parasite survival but simultaneously dampens the macrophage immune response, a phenomenon that is not well-explained by existing knowledge (19). Exosomal proteins such as GP63 and EF-1α function as immunosuppressive molecules and exacerbate *Leishmania* infection, and a recent study discovered a new type of exosomal molecule, *L. infantum* eukaryotic initiation factor (LieIF), which induces protective immunity (92). Both preinfection and postinfection applications of LieIF and IFN-γ promote NO and ROS production to inhibit *Leishmania* growth via a similar mechanism, although the former was correlated with the activation of macrophage inflammatory protein 1a (MIP-1a) and TNF-α, while the latter was not (24). The ability of LieIF to promote immune function and dampen *Leishmania* growth makes it a potential therapeutic molecule for leishmaniasis. In addition to proteins, non-coding RNAs (including rRNA, tRNA, and tRNA-derived small RNAs) were also found in exosomes secreted by *Leishmania braziliensis* and *L. donovani*, whereas siRNA was detected in only exosomes released by *L. braziliensis* (24). However, the functions of the non-coding RNAs in *Leishmania* exosomes remain unclear. In addition to producing exosomes in mammalian organisms, *L. infantum* and *L. major* were discovered to excrete exosomes in the midgut of sandflies via a mechanism resembling that in mammals (93). Exosomes and pathogens are coegested during biting by sandflies, probably accelerating the pathogenesis of leishmaniasis, especially cutaneous leishmaniasis. Coinjection of *L. major* and exosomes exacerbates inflammatory destruction, with increased levels of IL-17a and other inflammatory cytokines in the plasma. In *Leishmania* infection, IL-17a is involved in Th17 inflammation as well as neutrophil infiltration and leads to inflammatory lesions (94).

### EVs in Toxoplasmosis

Toxoplasmosis is a common parasitic disease caused by *Toxoplasma gondii*. Most patients have no clinical symptoms, while people with impaired immune systems may develop severe symptoms, such as severe damage to the fetus in pregnant patients or life-threatening encephalitis in immunocompromised patients (95). An animal model experiment showed that the injection of exosomes released by *T. gondii* antigen-stimulated DCs promoted the proliferation of splenocytes with enrichment in Th1 cytokines such as IL-2 and IFN-rs and reduced the expression of Th2 cytokines such as IL-4, IL-5, and IL-10 (25). Potent Th1-biased immune responses protect the host from severe *T. gondii* infection, which indicates the potential use of antigen-stimulated DC-derived exosomes in vaccine preparations (96). Further studies revealed that exosomes secreted by SRDCs cause a murine protective immune response against *T. gondii* infection with a strong cellular response and humoral response in mice, including the increased production of serum antibodies and IgA antibodies in the intestinal tract (97). Macrophages may also excrete corresponding exosomes when infected by intracellular pathogens, including *Salmonella typhimurium, M. tuberculosis, M. bovis* BCG, and *T. gondii*. Exosomes contain pathogen-associated molecular patterns (PAMPs), which represent a crucial mechanism for immune surveillance that functions in a Toll-like receptor (TLR)-dependent and myeloid differentiation factor 88 (MyD88)-dependent manner (26). Another study showed that one kind of exosome-like vesicle was released by human foreskin fibroblasts infected by *T. gondii*. These vesicles were characterized by abundant miRNAs and a significant increase in mRNAs relative to those of uninfected fibroblasts. The most enriched mRNAs included thymosin beta 4, eukaryotic elongation factor-1α (EF-1α), Rab-13, and LLP homolog, which were previously described to mediate neurologic activity. These results indicated that *T. gondii* exosomes probably mediate neurologic effects in toxoplasmosis (27).

In addition, it was previously found that host cells infected by *T. gondii* present altered cell cycles and cell proliferation (98). Neighboring cells such as L6 cells, a rat myoblast cell line, manifested a similar phenomenon, and transient retention at the S or G2/M cell phase when incubated with exosomes secreted by *T. gondii*-infected cells, which suggested an exosome-mediated cell cycle and cell proliferation. Further experiments were conducted to detect 64 miRNAs with signal intensity changes, 11 of which were supposed to regulate the expression of host cell genes such as cyclin D2 and retinoblastoma 1, which are related to chromosome movement, centrosome movement, sister chromatid segregation, and other functions (28).

Wowk et al. first identified *T. gondii*-derived exosomes and performed proteomic profiling on them. A wide range of classical exosome proteins were discovered, but the exact functions of *T. gondii* exosomes and their proteins remained unknown (99). Further studies discovered that *T. gondii*-derived exosomes had a mean size of 50 nm and consisted of several notable protein markers, including HSP70, CD63, and P30 (29). HSP70 and CD63 have been previously described as exosome markers (100), and P30 is known as a major surface marker of *T. gondii* (101), which indicates that these isolated vesicles can be designated *T. gondii*-derived exosomes. In addition, the exosomes promoted macrophage activation with increased production of IFN-γ, IL-12, and TNF-α; moreover, both humoral and cellular immune responses were stimulated in this process, resulting in protective immunity against *T. gondii* infection (29).

### EVs in Trichomoniasis

Trichomoniasis is a sexually transmitted disease caused by *T. vaginalis* with clinical genital symptoms such as itching, foul odor, and pain during sex or urination. Worse still, trichomoniasis can increase the incidence of HIV infection as well as prostate and cervical cancer. There are ~276 million new cases of trichomoniasis every year, and the disease mainly affects people who range from 15 to 49 years of age (102).

Previous studies have revealed three tetraspanins on the surface of *T. vaginalis* (103), and coincidently, tetraspanins are present in all mammalian exosomes and are used as a marker of exosomes (104). These results suggest that *T. vaginalis* may secrete exosomes as well. Olivia et al. first confirmed the existence of *T. vaginalis* exosomes and detected their composition, which not only included conserved exosomal proteins and RNA but also some specific parasite proteins (30). Adherence to epithelial cells is the first step for *T. vaginalis* to cause infection of the host (105). Exosomes released by highly adherent *T. vaginalis* were found to promote the adherence of poorly adherent species to epithelial cells. In addition to activating adherence, *T. vaginalis* exosomes may deliver regulatory molecules to the host and promote the modulation of host immune functions by inhibiting IL-8 secretion of ectocervical cells, consequently dampening neutrophil migration to infection sites (30). Another study demonstrated that exosome-like vesicles released by *T. vaginalis* were capable of inducing marked macrophage IL-10 production and a slight increase in IL-6 and TNF-α production. Pretreatment of these vesicles in a murine model also promoted production of IL-10 and inhibited the expression of immune cytokines such as IL-6, IL-13, and IL-17, suggesting that *T. vaginalis*-derived exosome-like vesicles modulate the host immune response and dampen inflammatory reactions (31).

### EVs in Trypanosomiasis

Trypanosomiasis is a parasitic disease caused by organisms in the *Trypanosoma* genus, including *Trypanosoma brucei*, and *Trypanosoma cruzi*, of which the former causes African trypanosomiasis or sleeping sickness, while the latter causes American trypanosomiasis or Chagas disease (106). Trypanosomes were discovered to deliver various types of vesicles to the host, modulating the host immune response and promoting parasite survival (33). Most studies investigating *Trypanosome* exosomes have focused on *T. brucei*. A previous proteomic analysis revealed that a large portion of proteins secreted by *T. brucei* lack transit peptides, implying an unusual protein secretion pathway in this parasite (33). Later, investigators found that *T. brucei* mainly excreted proteins via an exosome-based pathway. *T. brucei* exosomes were found to mediate the release of most excreted/secreted proteins, including abundant proteases that functioned in the pathogenic process and affected parasite nutrient supply, indicating the important role of exosomes in *T. brucei* infection (32, 33). In addition, for the *Trypanosoma* genus, mRNA formation involves the process of trans-splicing, which needs the assistance of a kind of RNA termed a spliced leader RNA (SL RNA). When trans-splicing was compromised in *T. brucei*, SL RNA accumulated and was released via an exosome-based pathway (107). These exosomes mediated parasite-parasite communication, and only intact exosomes influenced *Trypanosome* social motility. It was hypothesized that these exosomes functioned as repellents to keep the pathogens away from injured cells and improper environments to influence parasite social motility (34). Moreover, a series of studies showed that exosomes secreted by *T. brucei* were capable of modulating rRNA, snoRNA, and mRNA processing and quality control (35–37). The experimental data also suggested that *Trypanosome* exosomes are present mainly in the nucleus, according to the expression of exosome subunits RRP6 and RRP44 (37).

In recent years, *T. cruzi* exosomes have also been discovered. Researchers identified small RNAs in *T. cruzi* exosomes and found that they were mainly derived from tRNAs and rRNAs. tRNA-derived small RNAs (tsRNAs) colocalize with an Argonaute protein that is distinctive of trypanosomatids and can be passed on to other *T. cruzi* or host cells, which may promote transformation of the parasite from the epimastigote form to the trypomastigote form and increase host cell susceptibility (108, 109). Further studies have found that tsRNA carried by exosomes may induce changes in the expression profiles of some genes in host cells (38).

### EVs in Schistosomiasis

Schistosomiasis is caused by the *Schistosoma* genus (usually by *Schistosoma japonicum, Schistosoma mansoni*, or *Schistosoma haematobium*) and is one of the most important neglected tropical parasitic diseases worldwide. Schistosomiasis affects public health, second only to malaria, and sickens more than 200 million people and causes ~200 thousand deaths worldwide each year, especially in developing countries (110). The adult schistosome worm uses several immune-evasion strategies in the host and inhabits the mesenteric plexus in the portal system for a long time (111). Thus, detailed elucidation of the parasitism mechanism and biology of the schistosome are important for understanding the relationship between the host and schistosome, which may be useful for the identification of some novel biomarkers for schistosomiasis diagnosis and the development of new strategies to control schistosomiasis (112, 113). Recently, Wang et al. first isolated, identified and analyzed exosome-like vesicles derived from adult worms of *S. japonicum* and investigated their immune activities in macrophages. The results showed that the application of these vesicles prompted macrophage polarization into a classically activated form termed an M1 macrophage with increased production of pro-inflammatory factors such as TNF-α, CD16/32, and iNOS (39). Previous studies have demonstrated that the M1 macrophage plays an important role in killing parasites and preventing hepatic fibrosis (114). These results indicated that exosome-like vesicles secreted by adult worms of *S. japonicum* may be involved in activating the host immune response, which is different from the results of previous studies (39). Furthermore, 403 proteins in *S. japonicum* adult-worm-derived EVs were detected through proteomic analysis and found to possess the characteristics of catalytic activity, translation regulatory activity, binding activity and so on. In addition, these vesicles could also deliver miRNA to mammalian cells to mediate parasite-host interactions (40). Using the OptiPrep density gradient method, researchers have harvested a high yield of pure EVs in adult worms of *S. mansoni* and identified 130 schistosome proteins, as well as 143 miRNAs, some of which were detected in the sera of infected hosts (42, 115). In addition, to date, detecting eggs in stool or urine is still the “gold standard” for diagnosing schistosomiasis, although this detection is less sensitive in patients with a lower worm burden. On the other hand, using a serologic test increases the sensitivity but decreases the accuracy. Research by Meninger et al. provided a new diagnostic tool in patients with a low parasitic burden by detecting schistosome-specific miRNA, such as miR-2c-3p and bantam, isolated from EVs in sera from infected patients (43). Moreover, formerly DC-derived exosomes were found to modulate the overactivation of immune reactions in autoimmune diseases (69), and *S. japonicum* soluble egg antigen (SEA) was able to suppress colitis development in a murine model (116). Recent studies showed that the intraperitoneal injection of DC-derived exosomes pulsed by SEA was useful for attenuating the severity and repressing the progression of mouse inflammatory bowel disease (IBD), with decreased levels of pro-inflammatory cytokines such as IL-17a, IFN-γ, IL-22, IL-12, and TNF-α and increased levels of the anti-inflammatory cytokine TGF-β. Due to their anti-inflammatory effects, exosomes may have potential applications as immunosuppressive drugs (41). All of these results confirmed that schistosomes can directly secrete EVs, which may play important roles in host-schistosome interactions and be useful tools in the development of vaccines, diagnostics and therapeutics.

### EVs in Lymphatic Filariasis

Lymphatic filariasis (LF) is caused by Filarioidea, including *Brugia malayi, Wuchereria bancrofti*, and *Brugia timori*, and is the most common vector-transmitted parasitic disease after malaria. LF is still a public health problem, affecting more than 120 million people in 74 countries to date (2016). Patients usually have no symptoms, but some of them may present with elephantiasis, a syndrome characterized by severe swelling of the arms, breasts, genitalia, or legs and affecting patients clinically and socially (117). However, although mass drug administration (MDA) has been widely used globally and has some effect on decreasing the infection rate, there is still no drug that is completely effective at killing adult filarial nematodes, which means there is still no useful cure for LF (118). Recently, an increasing number of studies have shown that EVs released from filaria may be involved in this process. Mostafa et al. first identified exosome-like vesicles secreted from the infective L3 stage of *B. malayi* by designating a set of proteins as exosome markers, such as actin, EF-1α, EF-2, Rab-1, and HSP70 (44). Furthermore, the vesicles were exosome-like because a proteomic analysis revealed that the proteins of *B. malayi*-derived vesicles and those of mammalian exosomes shared over 80% homology. Moreover, vesicles released by L3 *B. malayi* contained an abundance of small RNA species relative to those released by the adult stage of *B. malayi*, suggesting they played a more important role in early infection. When comparing miRNA expression, researchers discovered significantly increased expression of bma-let-7, bma-miR-9, bma-miR-1, bma-miR-92, and bma-miR-100b. Bma-let-7 was previously described to modulate the vertebrate immune response, suggesting that these vesicles regulate the host immune response through an miRNA-dependent pathway (119, 120). In addition, the internalization of exosome-like vesicles by macrophages can stimulate M1 macrophage polarization to regulate the host immune reaction (44). Recently, Hirunni H et al. found that the candidate site for EV release may be the excretory-secretory pore of microfilariae. Their results revealed quantitative and qualitative differences between male and female *Brugia* EVs and sex-specific differences in cargo consisting of immunomodulatory substances. Moreover, the results confirmed that inhibiting EV release may be a potential method to kill filaria (121).

### EVs in Fascioliasis

Fascioliasis, a common helminthic zoonosis caused by *Fasciola hepatica* or *Fasciola gigantica*, mainly burdens ruminants such as sheep and cattle while sometimes affecting human beings. It is a noticeable public health problem with 2.4–17 million cases every year worldwide, especially in Europe, the Americas, Africa and Oceania (122). The parasite encysts in the small intestine and enters into the abdominal cavity after penetrating through the intestinal wall. Subsequently, the parasites in the abdominal cavity can migrate into the liver and ultimately penetrate into bile ducts (123).

Marcilla A. et al. first reported the presence of helminth-derived exosome-like vesicles that were found to contain species-specific proteins, including cytoskeletal proteins (i.e., actin, tubulin, myosin, paramyosin, tropomyosin), glycolytic enzymes (i.e., enolase, aldolase, GAPDH, PEPCK), calcium-binding proteins (i.e., calmodulin, calponin), and nuclear proteins (histones and elongation factors), as well as stress-related proteins (i.e., HSPs), detoxifying enzymes such as peroxiredoxins and host proteins including metabolic enzymes, immunoglobulins and typical exosomal molecules such as CD19 in *F. hepatica*. In addition, these exosome-like vesicles were found to be internalized by intestinal cells in culture and to mediate parasite-host communications (46). Researchers have confirmed that in addition to proteins, some potential immune-regulatory miRNAs were enriched in EVs of *F. hepatica* (124). Further studies confirmed the presence of exosome-like vesicles and demonstrated that these vesicles were shed from the apical plasma membrane via an ESCRT-dependent MVB pathway that occurred in the *F. hepatica* tegumental syncytium (45). Furthermore, the package of exosome-like vesicle cargo was regulated according to changes in the environment, probably implying an adaptive mechanism for *F. hepatica* survival and host immunosuppression (45).

### EVs in Rodent Intestinal Nematode Infection

*Heligmosomoides polygyrus* is a kind of nematode parasite that mainly infects mice. It exclusively locates to the intestinal tract and promotes a Th2 immune reaction with immunosuppressive effects (125). It was discovered that *H. polygyrus* is capable of secreting miRNAs and Y RNAs through an exosome-based pathway. *H. polygyrus* exosomes contain various proteins such as HSPs and tetraspanins that are found naturally in mammalian exosomes. Local injection of these exosomes in a murine model inhibits the type 2 innate response and eosinophil activation, subsequently repressing the host immune response. In addition, an *in vitro* assay demonstrated that miRNAs and Y RNAs in exosomes internalized by epithelial cells dampen the activation of IL-33 (the alarmin receptor) and DUSP1 (the critical regulator of mitogen-activated protein kinase signaling) to inhibit immune reactions (47).

Further studies have compared the composition of exosomes secreted by *H. polygyrus* with those secreted by its murine host. Tandem mass spectrometry analysis showed a 9- to 62-fold increase in plasmalogen content and a relative lack of sphingomyelin and cholesterol in *H. polygyrus* exosomes. Biophysical analysis revealed that *H. polygyrus* exosomes consist of a stable and rigid membrane structure, although past studies have shown that decreased levels of sphingomyelin and cholesterol destabilize the membrane structure (126). These results suggested that plasmalogen might play an important role in increasing membrane rigidity and stability (127).

### EVs in Cystic Echinococcosis

Cystic echinococcosis, a chronic zoonotic disease that is globally distributed in most pastoral and rangeland areas of the world, is induced by larval cestodes belonging to the genus *Echinococcus*, such as *Echinococcus multilocularis* and *Echinococcus granulosus*. Cystic echinococcosis features an asymptomatic incubation period that can last many years. Humans may contract this disease not only by ingesting soil or food contaminated with *E. granulosus* eggs but also by the hand-to-mouth transfer of eggs. The first cystic echinococcosis patient was observed in 1862 (128). From then on, cystic echinococcosis has become a noticeable parasitic disease that imposes an immense economic burden in a large number of countries (129). Mar Siles-Lucas et al. first reported that there were parasite exosome-enriched EV fractions in fertile hydatid cyst fluid (HF) and isolated, purified and characterized these exosomes. Both parasite-derived proteins (including antigen-5, severin/gelsolin/villin lipid transport protein, alpha-mannosidase, and malate-dehydrogenase) and host-origin proteins (including carbonic anhydrase, fructose-bisphosphate aldolase, peroxiredoxin, hemoglobin alpha and beta, pyruvate kinase, serum albumin, and triose phosphate isomerase) were identified in the EVs according to a proteomic analysis; the EVs carried virulence factors (including highly immunogenic and tolerogenic antigens and peptidases) that were associated with cyst survival. The results were the first to demonstrate that EVs may play an important role in infection by tape worms (130). In addition, Guilherme B. dos Santos et al. described a proteomic analysis and comparison of hydatid fertile samples that involved several proteins isolated from both the parasite and host during infection with *E. granulosus* fertile and infertile cysts in *Bos Taurus* lungs, aiming to highlight possible mechanisms involved in cyst fertility or infertility (131). In addition, the researchers found large differences between the proteomes. Therefore, the findings provided clues about the possible existence of an arms race involving parasites and host responses.

During early and chronic infections by *Echinococcus* species, nitric oxide (NO) is an important molecule that plays a role in downregulating host immunity (132). In particular, according to a report by Yadong Zheng (48), Ago1 and Ago4 expression by murine macrophage RAW264.7 cells was increased after transfection by *E. multilocularis* miR-71 (emu-miR-71) mimics. Interestingly, 12 h after treatment with LPS and IFN-γ, the production of NO was reduced, although the expression of IL-10 was not altered. Moreover, another study reported that the parasite can secrete exosomes containing miR-71 (133). EVs containing miR-71 may exert an important effect on macrophages, which introduces a new concept consisting of the interplay between parasites and hosts. However, the function of exosomes in host-parasite interactions in cystic echinococcosis is just beginning to be elucidated and still needs further investigation.

### EVs in *Teladorsagia circumcincta* Infections

*Teladorsagia circumcincta* infection, a major cause of ovine parasitic gastroenteritis in temperate climatic regions, leads to considerable economic losses. Ovine parasitic gastroenteritis mainly occurs in temperate parts of the world. Patients contract the disease through contaminated fecal matter and show reduced weight and dehydration due to diarrhea. Recent research indicates a terrible phenomenon in which the anthelmintic resistance of *T. circumcincta* is significantly increasing due to the overuse or misuse of existing drugs. It is rather remarkable that the exact potential SNPs and mechanism of anthelmintic resistance need further investigation (134). Previous research has mainly involved sheep infected with *T. circumcincta* (133). Stear MJ et al. found that the important mechanism of pathogenesis in sheep infected with the nematode *T. circumcincta* was that the nematode can cause protein deficiency and a lower growth rate and thus lead to serious mucus production, hyperplasia and pepsinogenemia (135). T. Thomas Tzelos et al. isolated exosome-like EVs from excretory-secretory (ES) products secreted by fourth stage larvae (Tci-L4ES) of *T. circumcincta* and performed a preliminary proteomic characterization. In addition, the researchers found some specific proteins that were involved in structure, metabolism and other functions. Most importantly, in *T. circumcincta*-infected sheep, the research indicated that these proteins can be bound by IgA and IgG and are a potential key to vaccine development and production (49).

## The Clinical Applications of Parasite EVs

Studies of EVs have been emerging constantly since their discovery in the 1980s. However, studies on parasite-derived EVs have been conducted for only a few years, and thus there is still much to be explored regarding the applications of parasite-derived EVs, giving rise to a new round of studies. All cell types can secrete EVs, and EVs are naturally found in different kinds of body fluids such as saliva, blood, urine, CSF, and even in milk. EVs were previously considered a waste disposal system but were subsequently found to be involved in cell-cell communication, thereby demonstrating multiple applications (136). Studies investigating the applications of parasite exosomes have mainly concentrated on the diagnosis and treatment of parasite diseases. Currently, we know that EVs are rich in lipids, proteins, small RNAs, and DNAs, many of which are available as molecular markers. Exosomes possess several major advantages as diagnostic markers, including (1) stable structure, (2) high content in plasma, and (3) alternative abundance and constitution under diverse conditions. Previously, EVs were found to be capable of helping diagnose malignant diseases because EVs contained a variety of tumor-specific molecules and shuttled them between cells to mediate intercellular communication. Specific EV molecules can be used as diagnostic markers for tumors, including NY-ESO-1 for lung cancer, HER2 for breast cancer, GGT1 for prostate cancer, glypican-1 for colorectal cancer, and CA-125 for ovarian cancer (137). For ovarian cancer, various exosomal cargos can be used as diagnostic markers, including (1) surface molecules such as EpCAM and L1CAM, (2) proteases such as ADAM10 and ADAM15, (3) tetraspanins, (4) HSPs, and (5) miRNAs such as miR-214, miR-140, and miR-147 (138). Unfortunately, in clinical practice, there is still no application for EVs in diagnosing parasite infections, and only a few studies have focused on the diagnostic use of EVs in parasitic diseases. Currently, we know that parasite EVs consist of a variety of molecules such as proteins, lipids, miRNAs, and DNAs (139). The detection of proteins, miRNAs or DNAs from isolated exosomes could represent a possible method to help diagnose parasitic infections. A recent study compared miRNA isolated from circulating exosomes in schistosomiasis patients and healthy controls and found a significant increase in 4 schistosomal miRNAs, including bantam, and miR-2c-3p (43). Traditional diagnostics for schistosomiasis are limited in patients with a low pathogen burden or cannot be used for patient follow-up (140, 141), indicating that the detection of miRNA from exosomes may have potential use for diagnosing patients with a low parasite burden or for follow-up of therapeutic effects (43).

Exosomes released by both parasites and host cells exert immunomodulatory properties, which has promoted investigations into their clinical applications for the treatment of diseases; currently, these applications mostly involve protection against tumors (142). For example, many researchers have demonstrated that DC-derived exosomes can stimulate patients' immune responses and promote the eradication of melanoma and non-small cell lung cancer (143, 144). With regard to parasite exosomes, as we mentioned, mice immunized with exosomes and infected by nonlethal *P. yoelii* elicit IgG antibody production, inducing reticulocytosis, and a protective immune response against malaria (15). *T. gondii*-derived exosomes may promote macrophage activation with increased production of IFN-γ, IL-12, and TNF-α; moreover, both humoral and cellular immune responses are stimulated in this process, resulting in protective immunity against *T. gondii* infection (29). Host cell-derived exosomes also execute immunoregulatory functions. For instance, DCs pulsed with *T. gondii* antigens induce the proliferation of splenocytes with Th1 cytokine enrichment and reduce the expression of Th2 cytokines, probably serving as a potent vaccine against toxoplasmosis (25). In addition to protecting against human parasitic infections, exosomes were discovered to induce a protective immune response against livestock parasites. Immunization of exosomes derived from *Eimeria* parasite antigen-loaded DCs protected chickens against avian coccidiosis, including *Eimeria tenella, Eimeria maxima*, and *Eimeria acervulina* infections, manifesting as symptom amelioration and mortality rate reduction (145).

Moreover, recent studies have revealed some new applications of exosomes in parasitic diseases. miRNAs in exosomes from the plasma of *P. yoelii* infectors inhibit VEGFR2 expression in endothelial cells and suppress regional angiogenesis, ultimately repressing the growth of Lewis lung cancer in a murine model (16). Further studies investigating *P. yoelii*-derived exosomal miRNAs may provide new therapies against tumors. Another investigation showed that the intraperitoneal injection of DC-derived exosomes pulsed by *S. japonicum* SEA is useful in attenuating the severity and repressing the progression of mouse IBD with decreased levels of pro-inflammatory cytokines and increased levels of anti-inflammatory cytokines (41). Due to their anti-inflammatory effects, exosomes may have potential applications as immunosuppressive drugs.

## Conclusion

**Figure 1:**
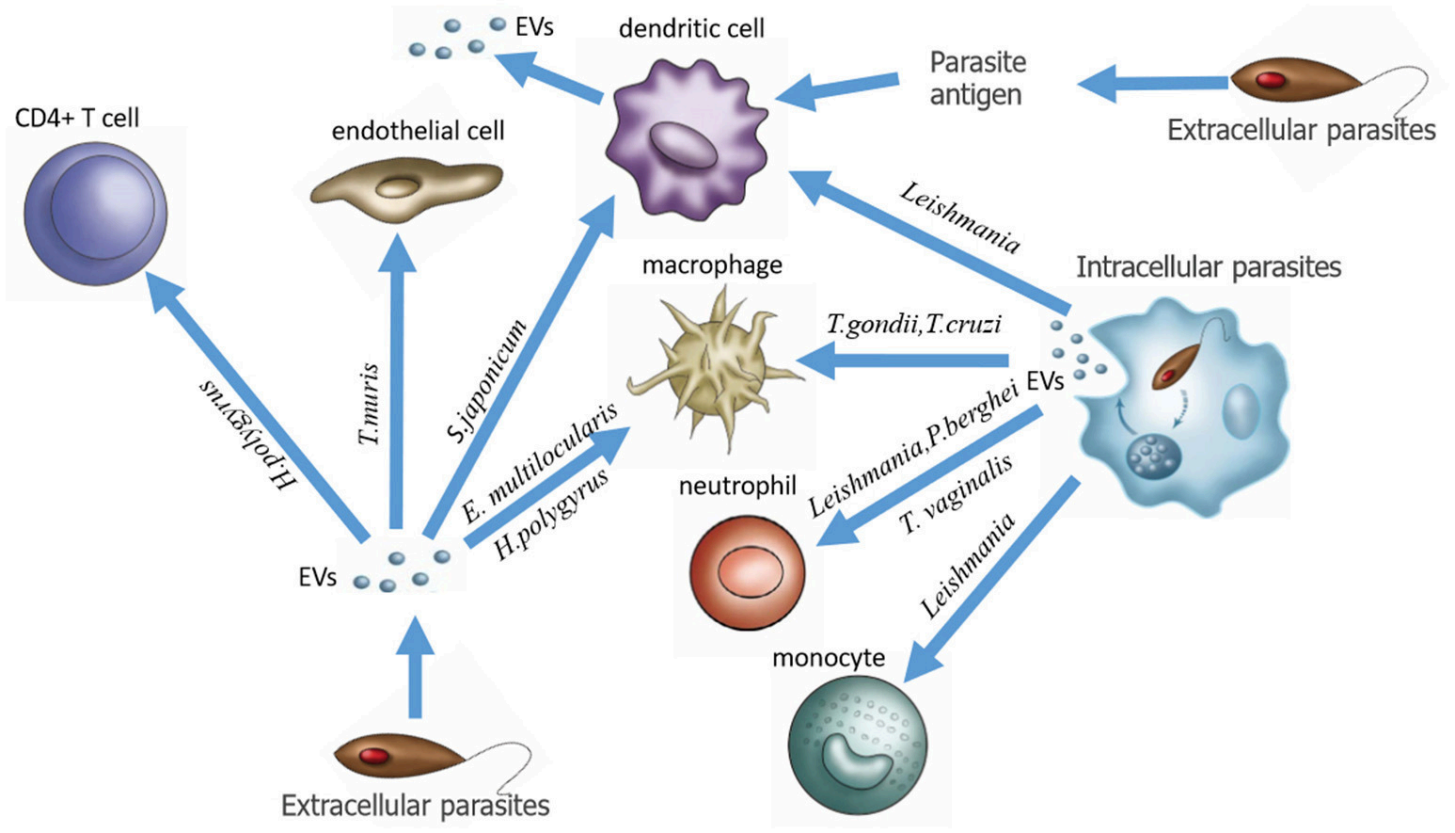
Three EV production modes associated with parasitic infection.

In this review, we give a general introduction to EVs and re-recognize the relationship between EVs and host-parasite interaction. Three groups of EVs are associated with parasitic infection: (1) EVs directly secreted from extracellular parasites, (2) EVs produced by host cells infected by intracellular parasites, and (3) EVs produced by host cells stimulated by parasite-derived antigens (Figure 1). Accumulating evidence indicates that parasite-derived EVs can act as signal molecules in parasite-host interactions to maintain normal parasitic physiology, leading to host pathogenesis. However, on the other hand, we should clearly recognize that EVs, as promising target drug delivery carriers, and useful biomarkers, have been extensively studied in other diseases such as cancer but still require more research in the context of parasitic infections. Although in recent years, a growing number of researchers have described the composition and function of EVs, the study of parasite EVs is still in its infancy, and the molecular mechanisms of EV formation, budding, and fusion as well as the function of EVs in host-parasite interactions remain to be further studied. Owing to the lack of consistent definitions and standards for the identification, isolation and analysis of EVs among laboratories, studies of EVs in different parasitic diseases seem somewhat confusing. We believe that after these problems are solved, more comprehensive studies of EVs will progressively reveal their exact roles in parasitic infections and provide new ideas and approaches for the application of exosomes in clinical practice.

## Author Contributions

XS and ZheW constructed the manuscript. ZhoW wrote the manuscript. JL and LinW collected the information. LifW revised the manuscript.

### Conflict of Interest Statement

The authors declare that the research was conducted in the absence of any commercial or financial relationships that could be construed as a potential conflict of interest.
